# 2220. High burden of antibiotic exposure in left ventricular assist device (LVAD) recipients: A call for enhanced antimicrobial stewardship

**DOI:** 10.1093/ofid/ofad500.1842

**Published:** 2023-11-27

**Authors:** William J Moore, Kendall Kling, Teresa Zembower, Alison Jirak, Gretchen Nonog, Grace Magliola, Rebecca S Harap, Jane Wilcox, Duc Pham, Tingqing Wu, Heather Byrd, Valentina Stosor

**Affiliations:** Northwestern Medicine, Chicago, Illinois; Northwestern University, Chicago, Illinois; Northwestern University, Chicago, Illinois; Northwestern University, Chicago, Illinois; Northwestern University, Chicago, Illinois; Northwestern University, Chicago, Illinois; Northwestern Memorial Hospital, Chicago, Illinois; Northwestern University, Chicago, Illinois; Northwestern University, Chicago, Illinois; Northwestern University, Chicago, Illinois; Northwestern University, Chicago, Illinois; Northwestern University Feinberg School of Medicine, Chicago, Illinois

## Abstract

**Background:**

LVAD patients are at high-risk for complications including hospitalization and infection, resulting in increased empiric use and lower thresholds for initiation of antibiotics (ABX). ABX utilization and risk for resistance development in this vulnerable patient population is not well established. We aimed to describe ABX exposure targeting early healthcare-associated infections (HAI) that occur in a complex patient population.

**Methods:**

This was a review of ABX use among a prospective cohort of patients with recent LVAD to assess antibiotic burden. Study patients are being followed longitudinally to assess microbiota changes that occur with duration of LVAD support, development of any LVAD-related and unrelated infection, and ABX exposure. Included patients were enrolled from 7/2022 to 3/2023. World Health Organization’s AWaRe criteria, which groups agents based on potential for resistance development and need for stewardship prioritization, was used to classify antibiotic use. Summary and descriptive statistics were performed.

**Results:**

Seventeen LVAD recipients were included with 53% having reported infection, totaling 21 distinct HAI. Infection types and antibiotic burden, including spectrum and duration are provided in Figure 1, with leading infection sites including respiratory (n=8), intra-abdominal (n=3), urine (n=3), and bloodstream (n=3). ABX courses exceeded most recommended durations, with a median of 11 days (IQR 8-16). Majority of episodes resulted in receipt of broad-spectrum antibiotics, including meropenem (n=7), piperacillin-tazobactam (n=6), and cefepime (n=4), despite limited occurrence of pathogens requiring these agents, *Enterobacter* spp (n=2), *Pseudomonas aeruginosa* (n=1). Observed ABX agents corresponded to WHO AWaRE Access (n=11.1%), Watch (n=83.3%), and Reserve (n=5.6%) groups, indicating frequent use of agents with high potential for resistance development.

**Figure 1.**
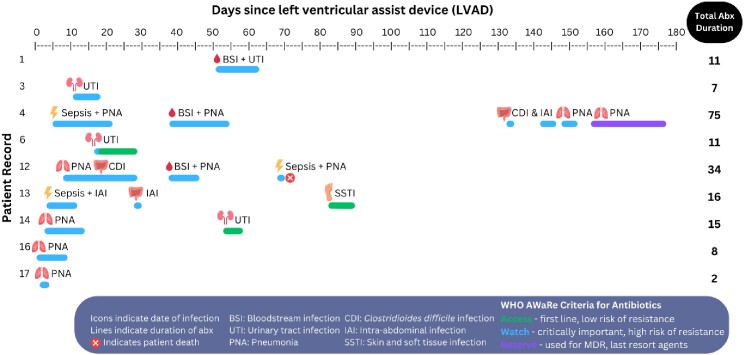
Antibiotic Burden & Infections following LVAD

Frequent use of broad-spectrum antimicrobials with high risk for resistance development observed in LVAD patients. WHO AWaRe criteria were used to identify higher risk agents, labeled as Watch and Reserve.

**Conclusion:**

We found high rates of early HAI and broad-spectrum antibiotic use in patients with recent LVAD placement. These findings demonstrate potential opportunities for enhanced antimicrobial stewardship in this population to optimize therapy selection, mitigate risk of subsequent multidrug-resistant infections, and to preserve essential agents for future use.

**Disclosures:**

**Valentina Stosor, MD**, DiaSorin S.p.A.: Advisor/Consultant|Eli Lilly & Company: Grant/Research Support

